# G2/M Cell Cycle Arrest and Tumor Selective Apoptosis of Acute Leukemia Cells by a Promising Benzophenone Thiosemicarbazone Compound

**DOI:** 10.1371/journal.pone.0136878

**Published:** 2015-09-11

**Authors:** Maia Cabrera, Natalia Gomez, Federico Remes Lenicov, Emiliana Echeverría, Carina Shayo, Albertina Moglioni, Natalia Fernández, Carlos Davio

**Affiliations:** 1 Instituto de Investigaciones Farmacológicas, Facultad de Farmacia y Bioquímica (ININFA-UBA-CONICET), Buenos Aires, Argentina; 2 Instituto de Investigaciones Biomédicas en Retrovirus y SIDA, Facultad de Medicina, (INBIRS-UBA-CONICET), Buenos Aires, Argentina; 3 Instituto de Biología y Medicina Experimental (IBYME-CONICET), Buenos Aires, Argentina; 4 Instituto de Química y Metabolismo del Fármaco, Facultad de Farmacia y Bioquímica, (IQUIMEFA-UBA-CONICET), Buenos Aires, Argentina; 5 Cátedra de Química Medicinal, Facultad de Farmacia y Bioquímica, Universidad de Buenos Aires, Buenos Aires, Argentina; Taipei Medical University, TAIWAN

## Abstract

Anti-mitotic therapies have been considered a hallmark in strategies against abnormally proliferating cells. Focusing on the extensively studied family of thiosemicarbazone (TSC) compounds, we have previously identified 4,4’-dimethoxybenzophenone thiosemicarbazone (T44Bf) as a promising pharmacological compound in a panel of human leukemia cell lines (HL60, U937, KG1a and Jurkat). Present findings indicate that T44Bf-mediated antiproliferative effects are associated with a reversible chronic mitotic arrest caused by defects in chromosome alignment, followed by induced programmed cell death. Furthermore, T44Bf selectively induces apoptosis in leukemia cell lines when compared to normal peripheral blood mononuclear cells. The underlying mechanism of action involves the activation of the mitochondria signaling pathway, with loss of mitochondrial membrane potential and sustained phosphorylation of anti-apoptotic protein Bcl-xL as well as increased Bcl-2 (enhanced phosphorylated fraction) and pro-apoptotic protein Bad levels. In addition, ERK signaling pathway activation was found to be a requisite for T44Bf apoptotic activity. Our findings further describe a novel activity for a benzophenone thiosemicarbazone and propose T44Bf as a promising anti-mitotic prototype to develop chemotherapeutic agents to treat acute leukemia malignancies.

## Introduction

Acute Myelogenous Leukemia (AML) comprises a group of hematological malignancies characterized by increased myeloid progenitor cells in bone marrow and/or peripheral blood. These cell subpopulations not only present diverse stages of hematopoietic differentiation, but also exhibit defects on the tightly controlled self-renewal process and failure in normal programmed cell death [1–3].

Currently, the treatment of AML is mainly based on the administration of therapeutic agents targeting DNA. Standard chemotherapy involves the combination of cytosine arabinoside (cytarabine) with an anthracycline, such as daunorubicin or idarubicin, or the anthracenedione mitoxantrone [4–6], whose underlying mechanism of action relies on neoplastic cell apoptosis [7, 8]. Alternative combinatorial approaches include agents like etoposide or doxorubicin, which induce DNA damage by topoisomerase II inhibition [9]. Such chemotherapeutic agents cause disruption of mitotic progression and prolonged activation of the mitotic checkpoint, mainly in p53-deficient tumor cells, which in turn leads to programmed cell death.

These strategies allow to reach complete remission rates of 50 to 75% in adult patients between 20 and 60 years old, although nearly 70% of these patients relapse or develop resistance to treatment [5]. In addition, many patients also suffer therapy-related complications such as elevated systemic toxicity and multidrug resistance. With the aim of diminishing chemotherapic resistance and the serious side effects caused by conventional treatments, a great effort is done in searching for new agents for AML treatment.

Thiosemicarbazones (TSCs) are a structurally diverse family of compounds that have been extensively studied because of their broad spectrum of pharmacological applications. Several reports have described their antibacterial [10, 11], antiprotozoal [12, 13] and antiviral activity [14], including, for instance, methisazone (Marboran), which is commercialized for smallpox treatment [15, 16]. Also, numerous compounds belonging to the thiosemicarbazone family have been examined both *in vitro* and *in vivo* for cytotoxic activity against several cancer types [17, 18]. The best characterized example is 3-aminopyridine-2-carboxaldehyde thiosemicarbazone (3-AP, also called Triapine), which has recently been included in clinical trials for cervical, colon and metastatic renal cancer treatment [19–22]. More recently, the heteroaromatic compound TSC S115 showed a broad antineoplastic activity and exerted synergistic apoptotic effects when used in combination with standard cytotoxic agents both *in vitro* and *in vivo* [23]. Although TSCs with antiproliferative activity exhibit a wide structural diversity, most of them share a mechanism of action associated to ribonucleotide reductase and topoisomerase II Alpha inhibition [24], reactive oxygen species generation and DNA damage [25–27]. Further supporting these mechanisms of action, other studies have demonstrated that TSCs can act as transition metal chelators and induce redox intracellular imbalance [28, 29].

In the search of new potential anti-leukemic drugs, a series of aromatic TSCs were previously synthesized in our laboratory and tested for antiproliferative activity in the U937 human acute leukemia cell line (unpublished data). From this biological screening, 4,4’-dimethoxybenzophenone thiosemicarbazone (T44Bf) was identified as the lead compound showing the most potent antiproliferative activity.

In the present work, we extended the evaluation of T44Bf to a panel of human acute leukemia cell lines (HL60, U937, KG1a and Jurkat) and described the mechanism underlying its antiproliferative effects. Our results show that T44Bf induced selective apoptosis by chronic mitotic arrest in these leukemia cell lines. Moreover, T44Bf-induced apoptosis involved mitochondrial membrane potential loss, sustained phosphorylation of anti-apoptotic protein Bcl-xL, and increased Bcl-2 with the observation of phosphorylated fraction. Also, we found that ERK signaling pathway upregulation was a requisite for T44Bf-induced cell death. Our findings further suggest that T44Bf acts as an anti-mitotic compound delaying anaphase onset by defects in chromosome alignment at prometaphase. In summary, T44Bf is a promising pharmacological prototype for the development of chemotherapeutic agents in the treatment of acute leukemia malignancies.

## Material and Methods

### 2.1 Reagents and antibodies

T44Bf was solubilized as a stock solution at 50 mM in dimethyl sulfoxide (DMSO) and stored at -20°C until use; for each experiment the final concentration of DMSO did not exceed 0.1%. Cell culture medium RPMI-1640 and antibiotics were obtained from Sigma Chemical Company (St. Louis, MO) and FBS from Natocor (Argentina). Anti-poly (ADP-ribose) polymerase (PARP), anti-Bcl-2, anti-Bax, anti-Bad, anti-Bcl-xL, monoclonal anti-pERK 1/2, anti-Cdc2 p34 (C-19), anti-Cyclin A, anti-Cyclin B1 and anti-αTubulin antibodies were purchased from Santa Cruz Biotechnology (Santa Cruz, CA, USA) whereas anti-ERK (clone MK12) antibody from Millipore (Merck KGaA, Darmstadt, Germany) and anti-caspase 3 antibody from Neuromics (Edina, MN, United States). A horseradish peroxidase-conjugated goat anti-mouse and anti-rabbit were used as the secondary antibody (Vector and Santa Cruz Biotechnology, respectively). Alexa-Fluor555 goat anti-rabbit IgG was from Invitrogen, Carlsbad, CA USA.

DiOC_6_ (3,3′-Dihexyloxacarbocyanine iodide), Hoechst, Paclitaxel, Oxaliplatin and anti-β actin antibody were purchased from Sigma Chemical Company (St. Louis, MO) and annexin V-FITC/PI apoptosis detection kit was obtained from BD Biosciences Pharmingen (San Diego, CA, USA). Other chemicals used were of analytical grade and were obtained from standard sources.

4,4’-dimethoxybenzophenone thiosemicarbazone (T44Bf) was prepared and characterized as previously described [30] by condensation of 4,4’-dimethoxybenzophenone with thiosemicarbazide. Both reagents were purchased from Sigma Chemical Company (St. Louis, MO).

### 2.2 Cell culture and synchronization

Human leukemia cell lines U937, HL60, KG1a and Jurkat were obtained from American Type Culture Collection (ATCC) and grown in RPMI-1640 medium (Sigma Aldrich Co.) supplemented with 10% fetal bovine serum (FBS) and 50 μg/ml Gentamicin in a humidified 5% CO_2_ atmosphere at 37°C.

Peripheral blood mononuclear cells (PBMC) were obtained from heparinized samples of healthy donors isolated by centrifugation on Ficoll-Hypaque. Cells were cultured at 37°C in a humidified atmosphere with 5% CO_2_ in RPMI-1640 medium, supplemented with 10% FBS and 50 μg/ml Gentamicin. For activation of the peripheral T cells, 2x10^6^ cells/ml were incubated with phytohemagglutinin A at a concentration of 1.0 μg/ml for 48h before treatment with T44Bf. Blood samples from normal volunteers were obtained after written informed consent in accordance with the Declaration of Helsinki. These studies were approved by the institutional review board of the National Academy of Medicine of Buenos Aires.

For cell synchronization at G0/G1, cells were serum-starved for 7h at 37°C and thereafter relieved into cell cycle by addition of 10% FBS. Before seeding, viability of cell lines and PBMC were tested by Trypan Blue assay. Cells were used only if viability was higher than 90%.

### 2.3 MTS assay

Cell proliferation was determined by a colorimetric assay using CellTiter 96 AQueous Non-Radioactive Cell Proliferation Assay (Promega, USA) according to the manufacturer’s instructions. For MTS assay, cells growing in exponential phase were seeded at 2.0x10^4^ cells/well in a 96-well plate and incubated in an atmosphere of 5% CO_2_ at 37°C. Cells were exposed to serial dilutions of T44Bf (0.78 μM to 50 μM) or 0.1% (v/v) DMSO (vehicle control group). After incubation for 48h, 20 μl of MTS was added to each well and further incubated for 2h at 37°C. The absorbance was measured at 490 nm using the FlexStation 3 microplate reader (Molecular Devices Inc., USA).

Half maximal inhibitory concentration 50 (IC_50_) values were calculated with GraphPad Prism software (GraphPad Software Inc., USA) using the sigmoidal dose-response function. Assays were carried out in triplicate and at least three independent experiments were conducted.

### 2.4 Analysis of cell cycle phases distribution by flow cytometry

Synchronized cell populations were treated with different concentrations of T44Bf or 0.05% (v/v) DMSO (vehicle) for 15h. After treatment, cells were harvested, washed with ice-cold PBS, fixed overnight by addition of 70% (v/v) ethanol and stored at -20°C for a minimum of 24h. On the day of flow cytometry analysis, cell suspensions were washed with ice-cold PBS and re-suspended in 50 μl RNaseA (100 μg/ml) at room temperature for 15 min.

Propidium Iodide was added to a final concentration of 20 μg/ml and incubated in dark at room temperature for 20 min. Cell cycle phase distributions were analyzed by FACS Scan Flow Cytometer (Beckton-Dickinson CA, USA). Data from at least three independent experiments were analyzed using ModFit software (VeritySoftware House Inc., Topsham, ME, USA) to determine the fractions of cells in the subG0/G1, G0/G1, S and G2/M phases from cell cycle distribution.

### 2.5 G2/M arrest reversion assay

Cells synchronized at G0/G1 and treated with 10 and 20 μM of T44Bf for 15h were washed with PBS and incubated in T44Bf-free culture medium for 2 and 5h. Flow cytometry analysis of cell cycle distribution was performed as previously described. Cells treated with Paclitaxel 250 nM served as positive G2/M arrest control.

### 2.6 Determination of apoptosis markers

#### 2.6.1 Annexin V binding assay

Cells growing in exponential phase were plated in 12-well plates at a density of 5.0x10^5^ cells/ml and cultured with different concentrations of T44Bf or vehicle (0.05% DMSO) in complete medium for 24h. After washing with ice-cold PBS, 2.0x10^5^ cells were incubated with FITC-labeled annexin V and PI according to the manufacturer’s instructions (BD Biosciences Pharmingen, San Diego, CA, USA) and analyzed by a FACS Scan Flow Cytometer (Becton-Dickinson CA, USA).

#### 2.6.2 Caspase 3 activity assay

Cells growing in exponential phase were seeded in 6-well plates and treated with different concentrations of T44Bf during 12 and 24h. Cells were then harvested and processed according to CASP3C caspase 3 colorimetric assay kit (Sigma Chemical Co. St. Louis, MO, USA). Absorbance at 405 nm, due to hydrolysis of the peptide substrate acetyl-Asp-Glu-Val-Asp p-nitroanilide (Ac-DEVD-pNA), was measured using the FlexStation 3 microplate reader (Molecular Devices Inc., USA) and caspase 3 activity was expressed as OD_405_ value.

### 2.7 Mitochondrial membrane potential evaluation (ΔΨm)

In order to assess T44Bf-mediated mitochondrial membrane potential in HL60 cells, 6x10^5^ cells/ml were seeded in 48-well plates and treated with 10 μM of T44Bf for 3, 5, 6 and 7h. After treatment, cells were harvested, centrifuged and incubated in the dark with 10 nM of the probe DiOC_6_ in RPMI-1640 for 20 min. Fluorescence was analyzed by flow cytometry (ʎ_ex_/ʎ_em_ = 488/530nm) using FACS Scan Flow Cytometer (Becton-Dickinson CA, USA) and results were analyzed with ModFit software (Verity).

### 2.8 Preparation of cell lysates and Western blot analysis

Cells were washed in PBS and lysed in 50 mM Tris–HCl pH 6.8, 2% SDS, 100 mM 2-mercaptoethanol, 10% glycerol and 0.05% bromophenol blue and sonicated to shear DNA. Cellular proteins from total cell lysates (20 μg) were electrophoresed on 8–15% SDS polyacrylamide gel and transferred to nitrocellulose membranes. Blots were blocked with 5% non-fat powdered milk in TBS containing 0.05% Tween-20 and probed with the indicated primary antibodies followed by horseradish-peroxidase-conjugated secondary antibodies. Reactivity was developed by enhanced chemiluminescence (ECL) according the manufacturer’s instructions (Amersham Life Science, England).

### 2.9 Immunofluorescence microscopy

Briefly, cell suspensions from each well were transferred to a microcentrifuge tube and fixed with 4% paraformaldehyde in PBS for 10 min. Cells were rinsed four times with cold PBS and permeabilized with 0.3% Triton X-100 in PBS before blocking non-specific binding sites with 10% v/v goat serum for 30 min. After 3 washes with PBS, cells were incubated with monoclonal anti-α-tubulin (1:50) overnight at 4°C followed by incubation with Alexa Fluor555 conjugated goat anti-rabbit (1:500) for 1h at room temperature. Specificity of the immunodetection was assessed by omitting the primary antibody. Nuclei were labeled by Hoechst staining and images were examined under the Eclipse E200, Nikon fluorescence microscope.

### 2.10 Statistical analysis

Results in Table 1 are expressed as the mean with a 95% confidence interval (IC_95_) and IC_50_ values calculated by the equation for sigmoidal dose-response using software Prism 5.00 for Windows. Other results are expressed as mean ± SD of at least three independent experiments. Statistical analysis was performed by Student´s t test or one-way ANOVA followed by Dunnetts´s or Student–Newman–Keuls (SNK) multiple comparisons post-test performed with GraphPad Prism 5.00 for Windows. A p-value of 0.05 or less was considered as statistically significant.

**Table 1:** Antiproliferative activity of T44Bf on different leukemia cell lines. Results are expressed as the concentration that induces 50% of cell proliferation inhibition (IC_50_) and the maximal inhibition achieved following 48h exposure to T44Bf. Data are presented as means and corresponding 95% confidence intervals (CI_95_) (n>3).

Cell line	IC_50_ μM (CI_95_)	Maximal Inhibition % (CI_95_)
Jurkat	4.2(3.8–5.1)	68.3(61.7–75.0)
U937	9.6(6.7–13.7)	96.2(81.1–104.2)
HL60	4.1(3.7–4.4)	88.9(84.7–91.6)
KG1a	6.4 (5.9–7.0)	61.1(55.8–66.3)

## Results

### 3.1 T44Bf inhibits proliferation by cell cycle arrest in human acute leukemia cell lines

**Fig 1:**
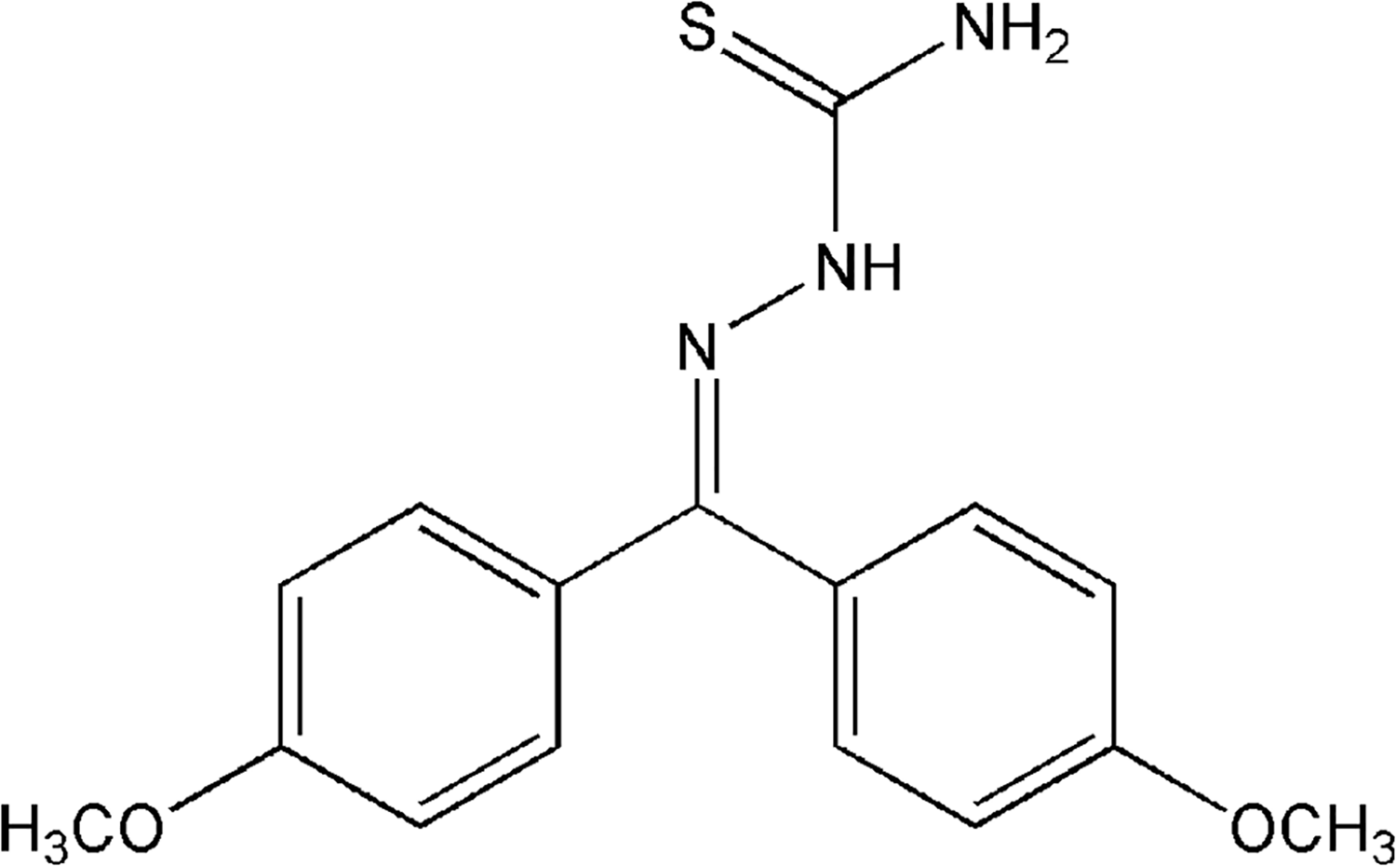
Chemical structure of 4,4’-dimethoxybenzophenone thiosemicarbazone (T44Bf).

In a previous screening of a series of aromatic thiosemicarbazones synthesized in our laboratory, we identified T44Bf (Fig 1) as a lead compound with potent antiproliferative activity in the U937 cell line (unpublished data). To further investigate the mechanism of action of T44Bf, we evaluated its activity on cell viability and proliferation in a wide panel of human acute leukemia cell lines with different stages of cell differentiation. Table 1 shows that treatment with T44Bf for 48h inhibited cell proliferation by approximately 90% in U937 and HL60 cells, and 60% and 70% in KG1a and Jurkat cells, respectively. In all cases, the half maximal inhibitory concentration (IC_50_) was in the low micromolar range supporting the high potency of T44Bf as a cell proliferation inhibitor in human acute leukemia cell lines.

**Fig 2:**
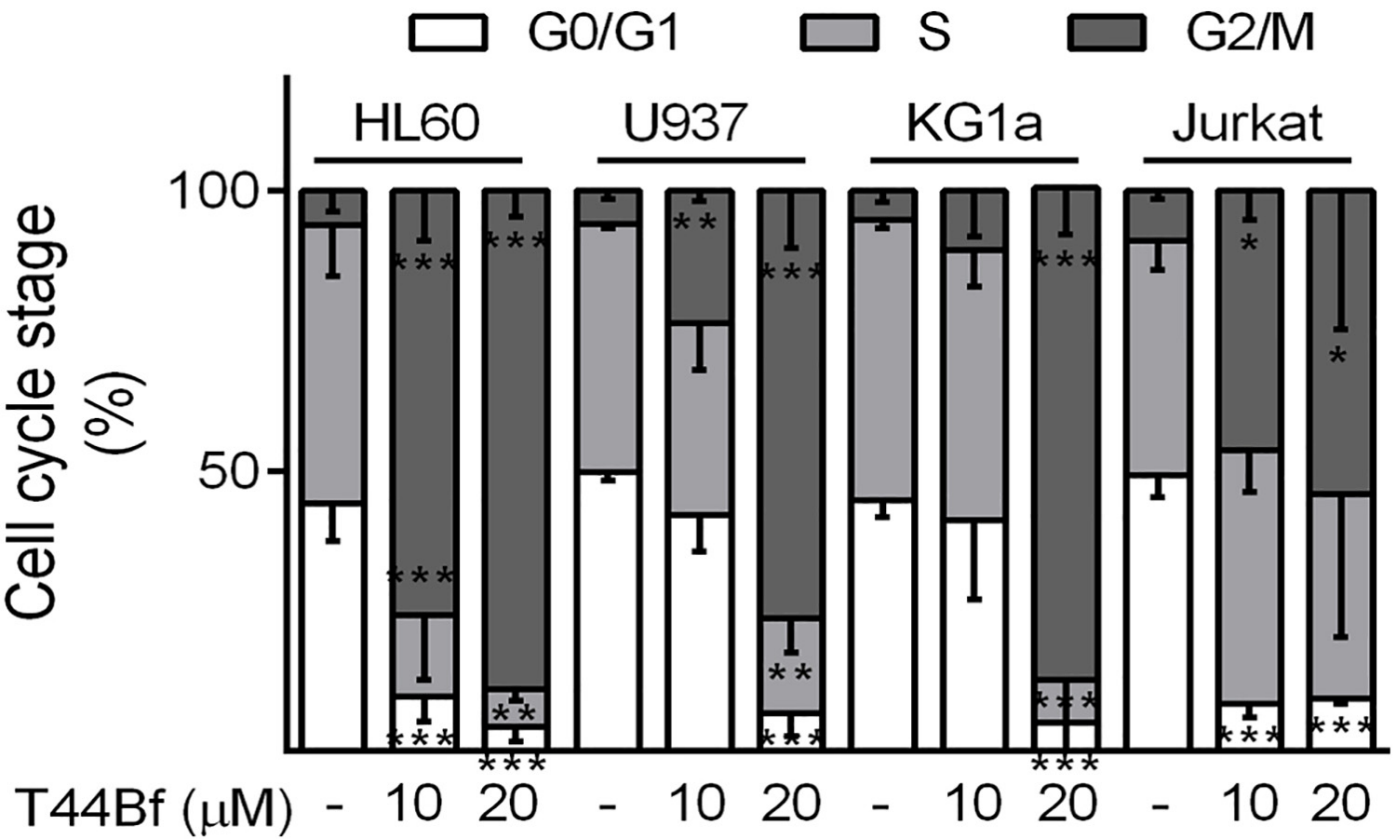
Cell cycle distribution after T44Bf treatment in human acute leukemia cell lines. Synchronized G0/G1 cells were exposed to T44Bf at the indicated concentrations or to 0.05% (v/v) DMSO, vehicle control group for 15h. Cell cycle distribution was calculated as described in Material and Methods. Data represent the mean ± SD (n > 3). *P < 0.05

In an attempt to evaluate whether T44Bf-mediated inhibition on cell proliferation correlated with effects on cell cycle progression, cell cycle distribution of T44Bf-treated cells in a concentration-response manner was assessed by flow cytometry. Synchronized cells exposed to 10 or 20 μM T44Bf for 15h showed a pronounced increase in the G2/M population with a concomitant reduction of cells in G0/G1 phase in all cell lines (Fig 2). In HL60 cells, T44Bf induced a significant G2/M arrest at both concentrations, however at 20 μM the subG0/G1 population was increased (data not shown). On the contrary, in U937, KG1a and Jurkat cells, T44Bf at 20 μM lead to a maximal arrest at G2/M when compared to 10 μM after 15h without changes in subG0/G1. These results indicate that the anti-proliferative effect of T44Bf is associated with an arrest in G2/M phase of the cell cycle.

### 3.2 T44Bf induces apoptosis selectively in human acute leukemia cell lines

We evaluated whether T44Bf exerted antiproliferative activity via the induction of apoptosis in addition to cell cycle arrest. Several key apoptosis-related events in response to T44Bf treatment, including phosphatidylserine exposure, cleavage of caspase 3 and poly (ADP-ribose) polymerase (PARP) and caspase 3 enzymatic activity were assessed. Given the differences observed in the maximal values of cell proliferation inhibition obtained from the MTS assay and knowing that multiple pathways may trigger the apoptotic response, apoptotic markers were assessed using 10 and 20 μM T44Bf, in an attempt to detect the maximal apoptotic activity.

**Fig 3:**
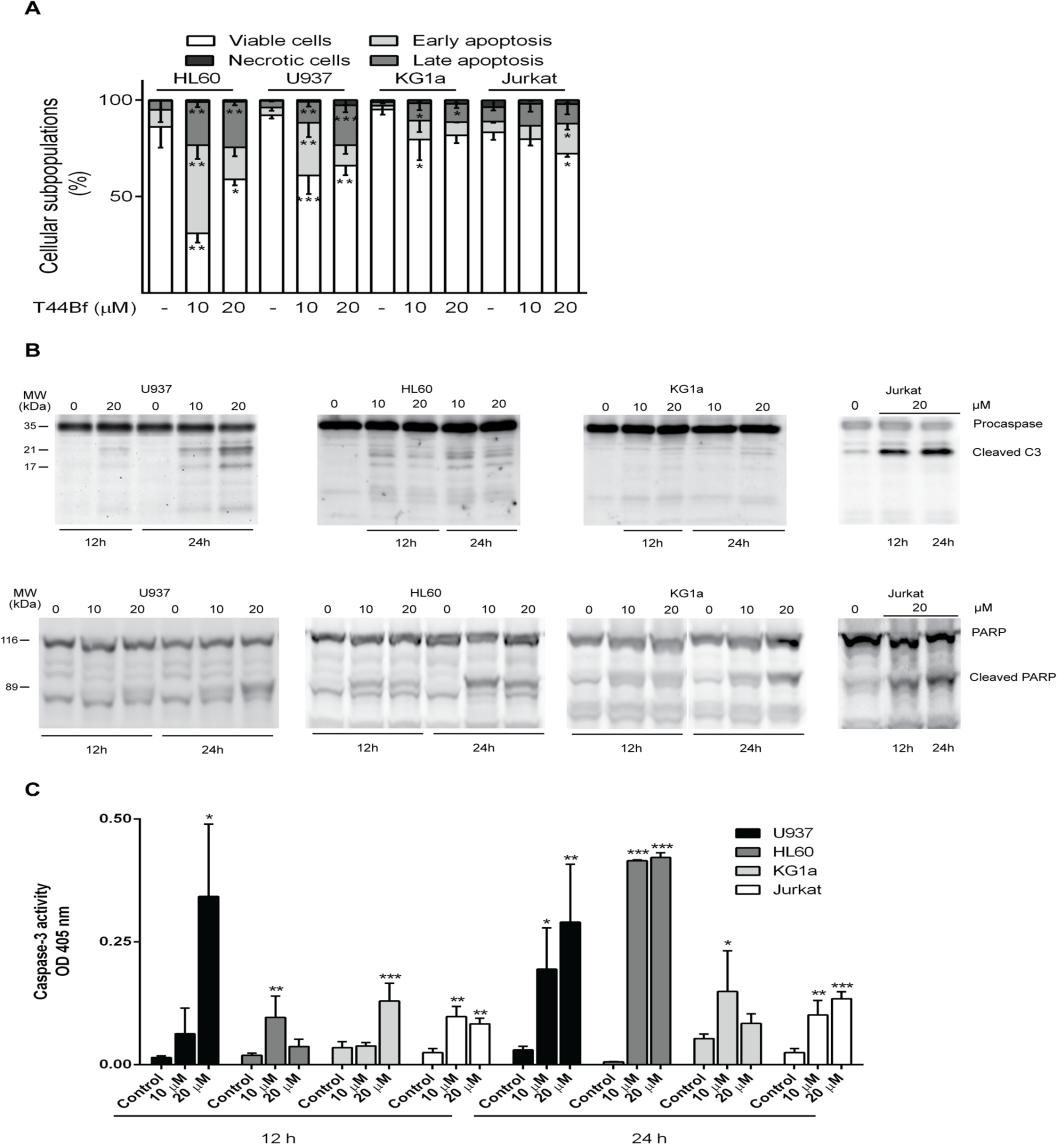
Pro-apoptotic activity of T44Bf in human acute leukemia cell lines. Cells in exponential growth were exposed to T44Bf at the indicated concentrations or to 0.05% (v/v) DMSO, vehicle control group. (A) After 24h of treatment cells were analyzed to detect exposed phosphatidylserine by annexin V binding assay. The graphic shows the different cell subpopulations according to the annexin V/PI staining pattern: cells labeled with only annexin V (early apoptosis), cells labeled with annexin V and PI (late apoptosis), and cells labeled only with PI (necrotic cells). (B) Determination of cleaved caspase 3 and PARP by Western blot. Equal amounts of protein were subjected to SDS–PAGE with anti-caspase 3 and PARP antibodies. Data are representative of at least three independent experiments. (C) Caspase 3 enzimatic activity induced by T44Bf in human acute leukemia cell lines. Cells were treated with T44Bf in the indicated concentrations and time, and caspase 3 protease activity measured as described in Materials and Methods and expressed as OD_405nm_ values. Data are presented as mean ± SD from four independent experiments. *P<0.05.

Annexin V/PI staining was performed in cells exposed to 10 and 20 μM for 24h. A significant increase in the early- and late-apoptotic populations was observed in HL60 and U937 cell lines at both T44Bf concentrations, whereas KG1a cells showed only a late-apoptotic population. In addition, while HL60 and U937 cells displayed the highest sensitivity to the treatment, Jurkat cells only showed a significant increase in the early-apoptotic population at 20 μM of T44Bf. It is worth noting that T44Bf did not promote necrosis at the times tested in any of the cell lines (Fig 3A).

Next, we assessed the cleavage of caspase 3, the terminal effector in the apoptotic cascade, and its substrate PARP by western blot. Cells incubated with T44Bf exhibited a time- and concentration-dependent increase in both active fractions of caspase 3 (Fig 3B upper panel) and in the 89-kDa fraction corresponding to cleaved PARP (Fig 3B lower panel). These findings strongly suggest that T44Bf induced caspase 3 activation, which was confirmed by enzymatic caspase 3 activity assessment using a colorimetric assay. Incubation with T44Bf augmented the enzymatic activity of caspase 3 in all cell lines, in agreement with the results obtained by western blot (Fig 3C). With the aim to compare all cell lines we unified the time of treatment and selected for further assays the concentration of 10 μM for HL60 cells and 20 μM for U937, KG1a and Jurkat cells.

These results clearly indicate that although the sensitivity of leukemia cell lines differ, all of them display a significant induction of apoptosis through the activation of the caspase pathway in response to low concentrations of T44Bf.

**Fig 4:**
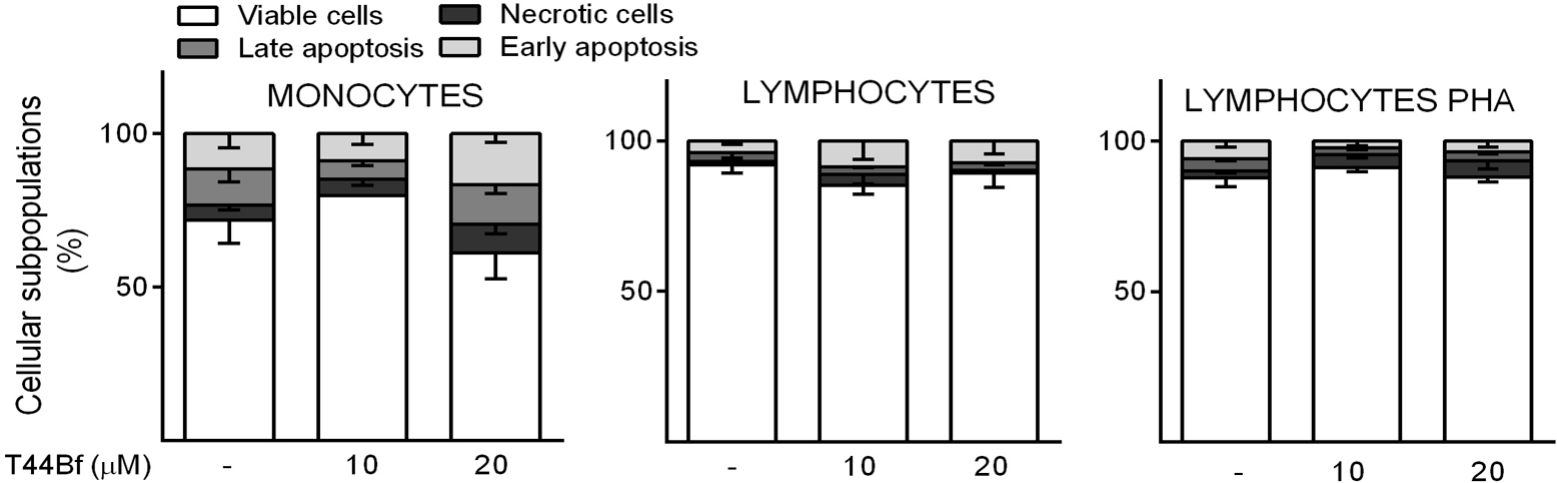
Compound selectivity assessed in normal peripheral blood mononuclear cells (PBMC). Phosphatidylserine exposure was measured by annexin V binding assay in monocytes, unstimulated lymphocytes or Phytohemagglutinin A activated (i.e. proliferating) lymphocytes after 24h treatment with T44Bf at the indicated concentrations or to 0.05% (v/v) DMSO vehicle control group. Data represent the mean ± SD (n > 3). *P<0.05.

In order to evaluate the selectivity of T44Bf for neoplastic cells, normal peripheral blood mononuclear cells (PBMC) were incubated with different T44Bf concentrations and viability was assessed by annexin V/PI staining. Interestingly, the T44Bf concentrations that induced apoptosis in leukemic cells failed to induce cell death in normal PBMC, as observed specifically in monocytes, unstimulated lymphocytes and Phytohemagglutinin A activated (i.e. proliferating) lymphocytes (Fig 4), thus supporting the therapeutic potential of T44Bf based on its selective pro-apoptotic effect and reduced toxicity.

### 3.3 T44Bf induces apoptosis in HL60 cells through the activation of the mitochondrial pathway

**Fig 5:**
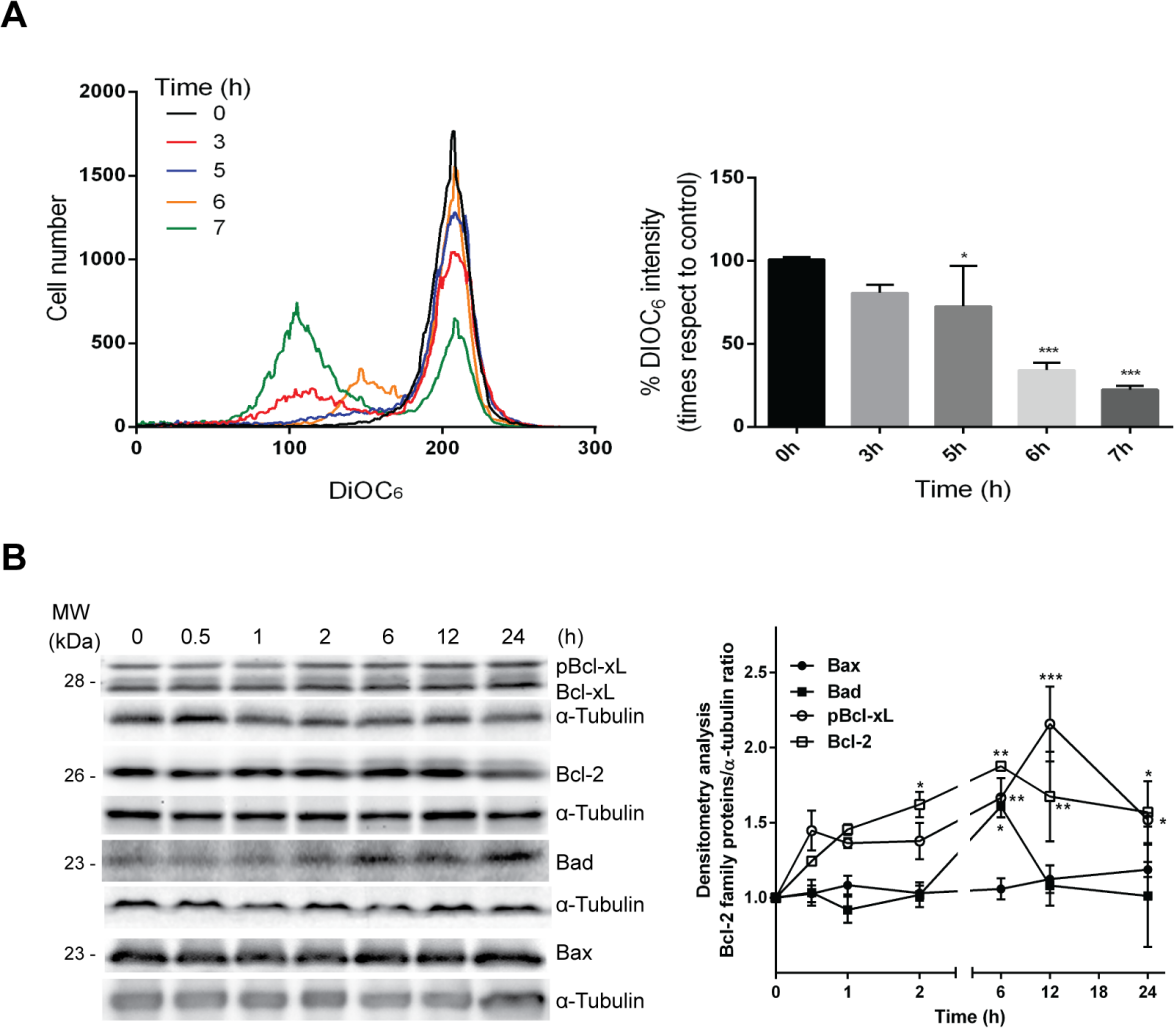
T44Bf effects on mitochondrial membrane potential and Bcl-2 family protein levels. (A) Cells treated for 3, 5, 6 and 7h with T44Bf or 0.05% (v/v) DMSO vehicle control group, were evaluated for changes in fluorescence intensity of DiOC_6_ probe by flow cytometry. The bar graph shows differences among treatment times. (B) The representative graphic shows Bcl-2, Bad, Bax and Bcl-xL protein assessment by Western blot. Equal amounts of protein were subjected to SDS–PAGE with anti-Bcl-2 family protein antibodies. Blots were subjected to densitometry analysis using ImageJ software. Data are presented as mean ± SD respect to control of at least four independent experiments. *P < 0.05

In order to gain further insight into the mechanism of apoptosis exerted by T44Bf on leukemic cells we performed a series of experiments in HL60 cells. We chose HL60 cells because they exhibit a low stage of differentiation and high sensitivity to T44Bf. Since mitochondrial damage represents an event extensively associated to apoptosis, we first evaluated the loss of mitochondrial membrane potential by measuring changes in DiOC_6_ fluorescence levels. DiOC_6_ accumulates inside intact mitochondria and therefore, a loss of DiOC_6_ fluorescence intensity implies damaged or leaky mitochondria membranes. Incubation of HL60 cells with 10 μM T44Bf decreased DiOC_6_ fluorescence indicating impairment of mitochondrial membrane integrity (Fig 5A). The change in the mitochondrial membrane potential was significant after 5h of T44Bf treatment, reaching the maximum at 7h with 75% of fluorescence loss. In accordance, changes in Bcl-2 and Bcl-xL, two proteins related to the maintenance of mitochondrial integrity were observed (Fig 5B).When the anti-apoptotic protein Bcl-xL was evaluated, results showed a sustained increase in the phosphorylated fraction following 6h exposure to T44Bf. Furthermore, a sustained increase in Bcl-2 expression after 2h was also observed. In addition, the immunoblotting showed a Bcl-2 mobility shifted band after 2h treatment which corresponds to the Bcl-2 phosphorylated fraction as reported by other groups [31, 32]. We also evaluated the pro-apoptotic proteins Bad and Bax, and found that only Bad increased at 6h whereas Bax remained unchanged at all times evaluated (Fig 5B). These results show that T44Bf induces cell death through the activation of the mitochondrial signaling pathway.

### 3.4 ERK1/2 activation is required for T44Bf-induced apoptosis

**Fig 6:**
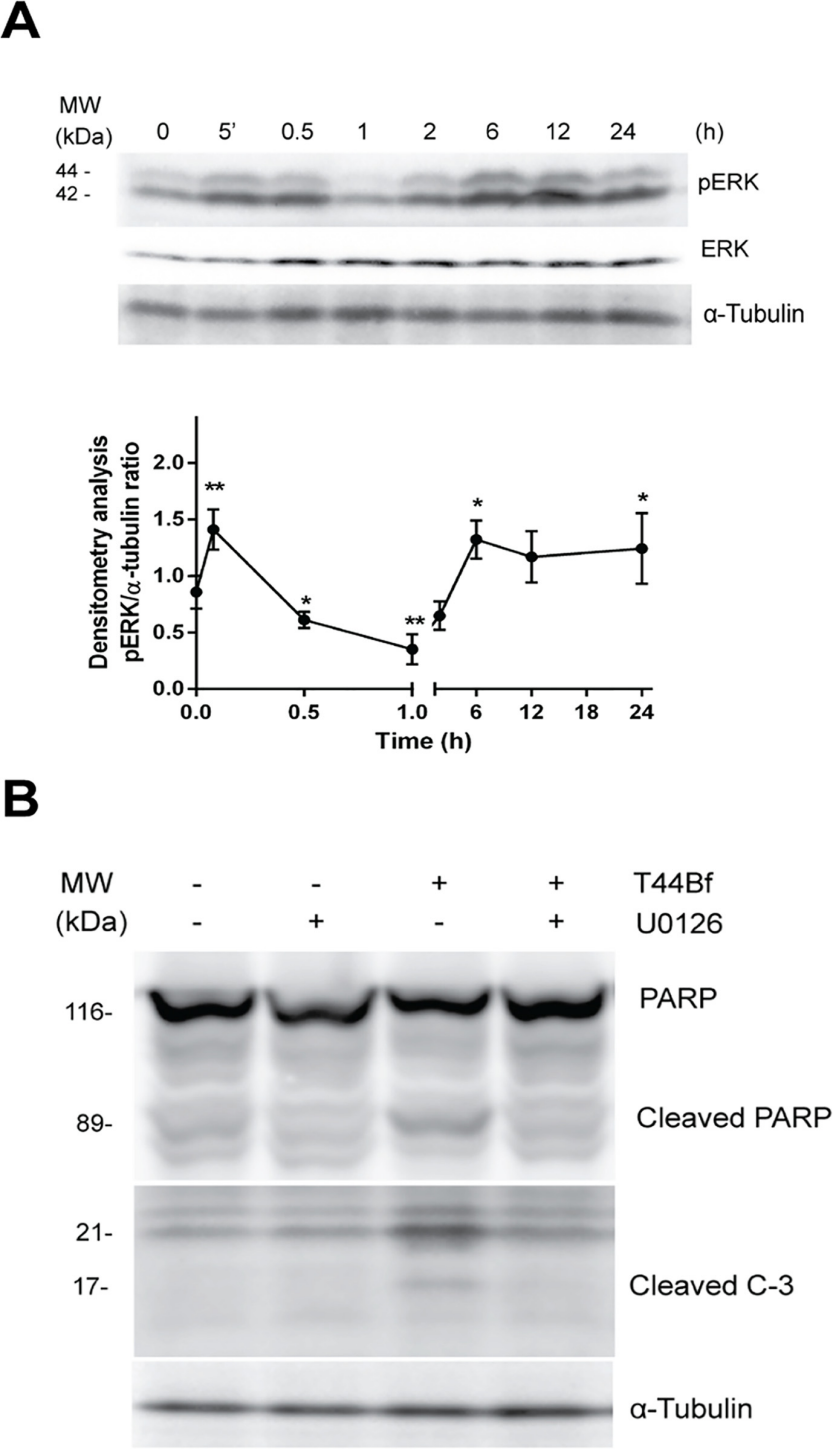
ERK time-course phosphorylation after T44Bf treatment. (A) Representative blot of ERK 1/2 phosphorylation following exposure of HL60 cells to 10 μM T44Bf at different times. Equal amounts of protein were subjected to SDS–PAGE with anti-pERK 1/2 antibody. Blots were stripped and incubated with ERK total antibody and α-tubulin as loading control. Bar plot showing arbitrary units obtained from densitometry measurement. Data are expressed as mean ± SD of three independent experiments. *P < 0.05. (B) Effect of MEK inhibitor U0126 on T44Bf-induced apoptosis after 12h in the HL60 cell line. Blot of cleaved caspase 3 and PARP after T44Bf treatment in the presence of 10 μM U0126. Graphic is representative of three independent experiments.

Several reports support that ERK phosphorylation is a relevant step in the chain of events leading to programmed cell death [33–35]. In this sense, prolonged activation associated with apoptosis has been shown for diverse cytotoxic compounds [36, 37]. Therefore to further elucidate T44Bf mechanism of action, we measured ERK phosphorylation in HL60 cells. Treatment with 10 μM T44Bf led to a rapid and significant increase in pERK levels at 5min, followed by a late phosphorylation at 6h and 24h (Fig 6A). Then, in order to determine whether MEK/ERK signaling was involved in T44Bf pro-apoptotic effect, we measured apoptotic markers in the presence of the MEK/ERK inhibitor U0126 (10 μM). Interestingly, under these conditions, the cleaved fractions of both PARP and caspase 3 proteins were not observed (Fig 6B), thus showing that the MEK/ERK pathway inhibition dampens the pro-apoptotic action of T44Bf.

### 3.5 T44Bf induces mitotic aberrations

**Fig 7:**
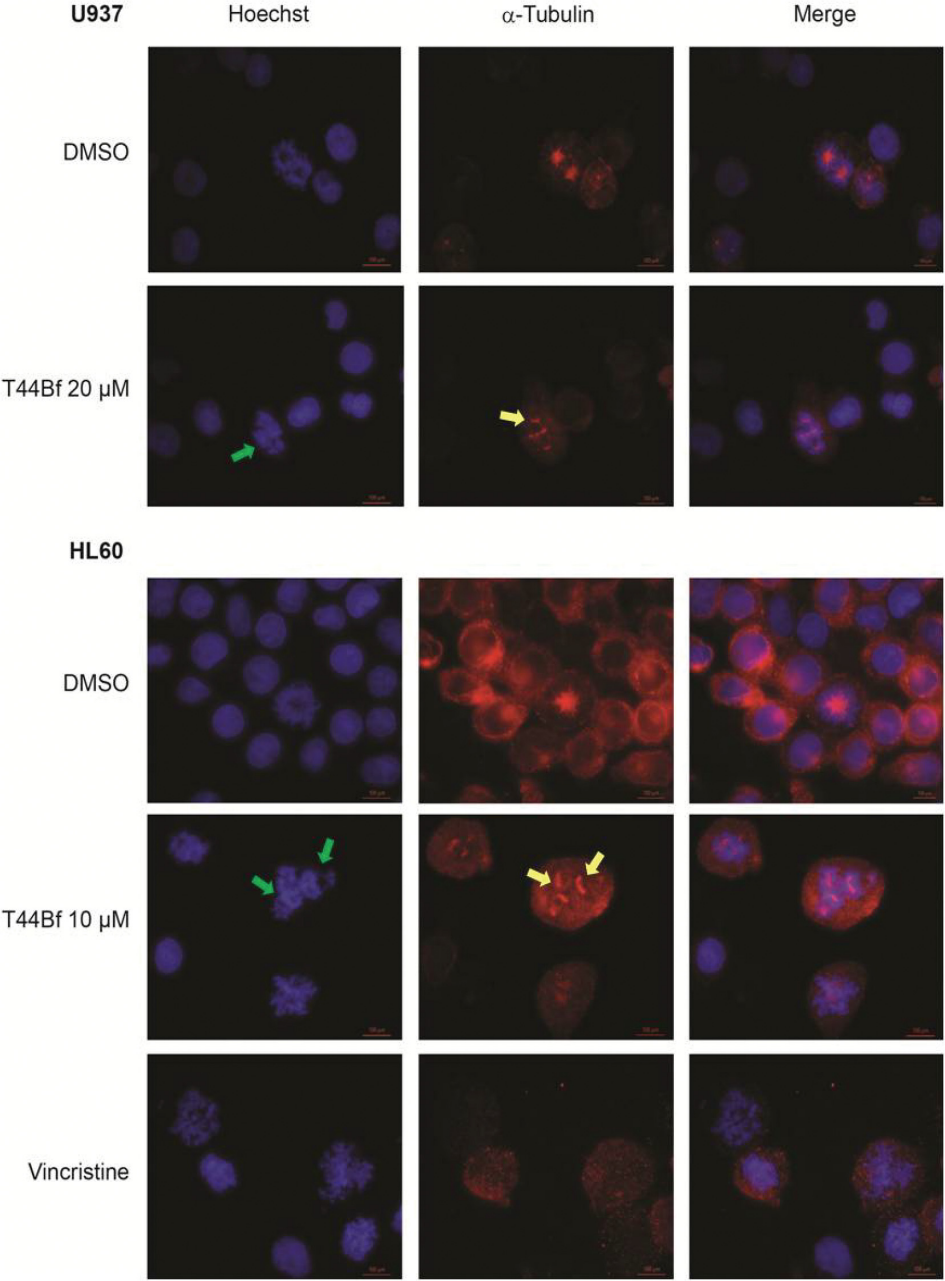
Indirect immunofluorescence microscopy in HL60 and U937 cells treated with T44Bf. Synchronized cells were treated with T44Bf (10 μM and 20 μM) or Vincristine (200 nM) for 15h. Cells were fixed and stained to detect α-tubulin (red) and counterstained by Hoechst for DNA (blue). Mounted slides were visualized under 1000X magnification on a Nikon Eclipse E200 fluorescence microscope. Yellow arrows indicate extra spindle poles and green arrows indicate unaligned chromosomes. Images are representative of three independent experiments.

Previous reports indicate that when disorders in the mitotic division occur, the apoptotic pathway is activated by a mechanism involving MEK/ERK signaling [36, 38]. Knowing that T44Bf causes cell cycle arrest in G2/M and induces apoptosis by the MEK/ERK dependent pathway, we aimed to identify the stage of mitosis targeted by T44Bf treatment. By visualization of the microtubule network through an immunofluorescence assay it was observed that HL60 and U937 cells treated with T44Bf showed morphological features associated with cells blocked at mitotic prometaphase [39]. Furthermore, treatment with T44Bf led to disorders in the mitotic process as evidenced by the appearance of extra spindle poles (Fig 7). In addition, DAPI staining revealed the presence of chromosomes which failed to localize completely at the equator of the mitotic spindle. A similar profile in chromosome distribution was obtained in HL60 cells treated with Vincristine (200 nM) [40], which was used as a positive control of prometaphase arrest. Overall, these results validate the cell cycle arrest observed by flow cytometry and support the idea that T44Bf treatment blocks cell cycle in prometaphase. These findings further suggest that T44Bf may affect the proper organization of the mitotic spindle and/or the proper alignment of chromosomes.

### 3.6 T44Bf-induced prometaphase arrest is a reversible event

**Fig 8:**
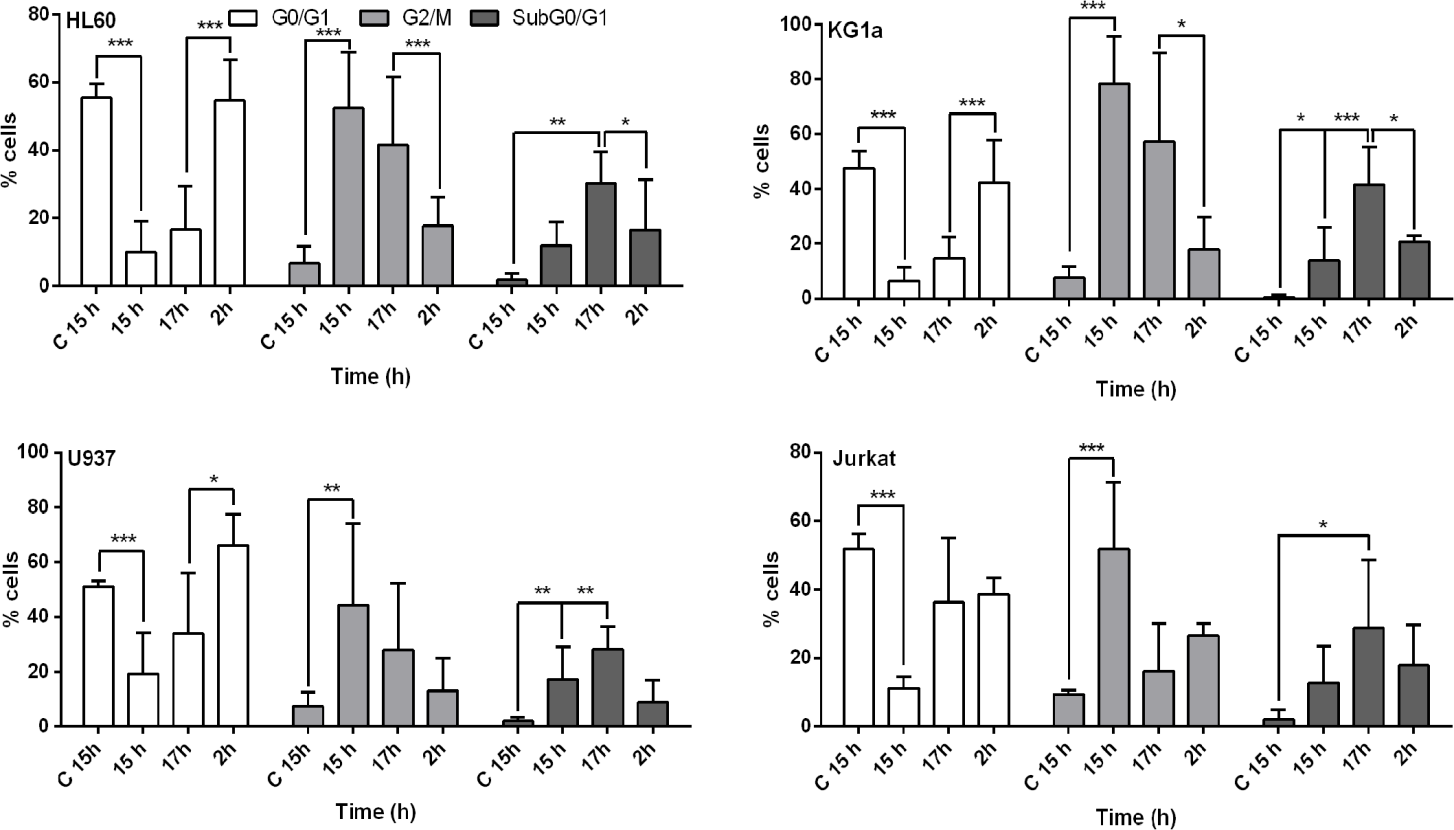
Cell cycle progression after T44Bf removal. Cells were exposed to 10 (HL60) and 20 μM (U937, KG1a and Jurkat) of T44Bf for 15h. Then, the drug-containing medium was removed and replaced by fresh culture medium for 2h. Cells were stained with PI and analyzed by flow cytometry. Data are expressed as mean ± SD of three independent experiments. *P<0.05.

With the aim to determine a time window for T44Bf activity we evaluated whether the observed alterations in cell cycle progression could be reverted. After 15h treatment, T44Bf was replaced by fresh medium and cell cycle distribution of leukemic cells assessed. Interestingly, 2h after T44Bf removal, the levels of G0/G1 subpopulation were restored to values similar to control cells in HL60, U937 and KG1a cell lines (Fig 8). However, lymphoid Jurkat cells did not exhibit significant differences in the cell cycle distribution following T44Bf removal. When the subG0/G1 subpopulation was evaluated under the same conditions, HL60 and KG1a cells showed significant differences as compared to cells exposed to T44Bf. These findings clearly indicate that T44Bf-induced cell mitotic blockage is reverted when the compound is washed-out, allowing cells to transit to G0/G1 phase and therefore emphasize the relationship between cell cycle arrest and apoptosis.

### 3.7 T44Bf-induced prometaphase arrest is associated to increased levels of Cyclin B1 and downregulation of Cyclin A

**Fig 9:**
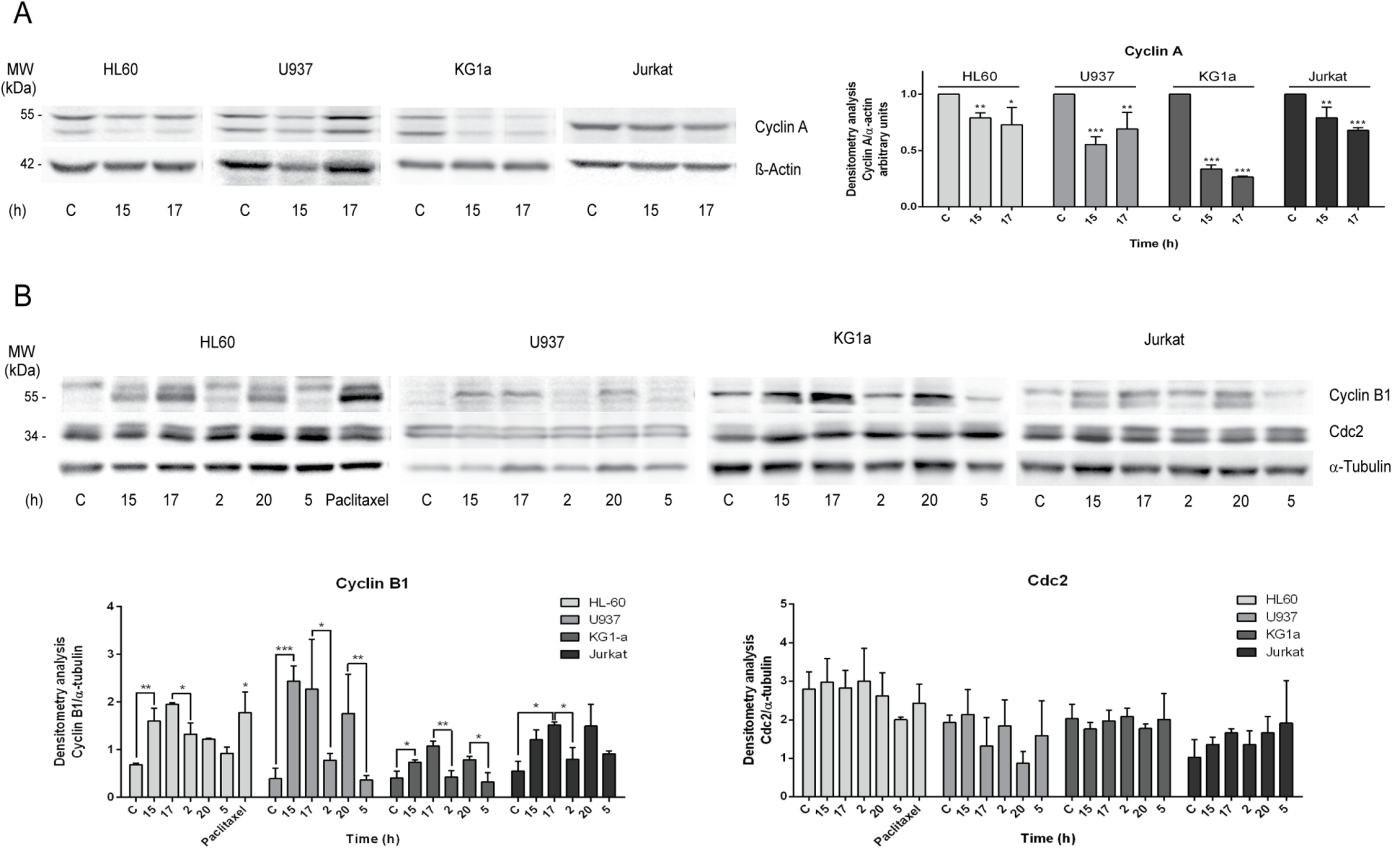
Cyclin A, Cyclin B1and Cdc2 evaluation by western blot. (A)Total lysates of cells exposed to 10 (HL60) and 20 μM (U937, KG1a and Jurkat) T44Bf for 15 and 17h were analyzed for Cyclin A by Western blot. Densitometry for Cyclin A blot was performed using Image J software. Arbitrary units represent protein level analysis respect to load control β-actin. (B) Cells were exposed to 10 (HL60) and 20 μM (U937, KG1a and Jurkat) T44Bf for 15h. Then, the drug-containing medium was replaced by fresh culture medium for 2 and 5h. Total cell lysates were analyzed by Western blot for Cyclin B1 and Cdc2. Densitometry for Cyclin B1 and Cdc2 blots were performed using Image J software. Arbitrary units represent protein level analysis respect to load control α-tubulin. *P < 0.05.

To gain further insight into T44Bf-induced prometaphase arrest, we next examined the effect on mitotic regulatory proteins such as Cyclin A, Cyclin B1 and Cdc 2. Cyclin A is a protein associated to cell cycle progression. It rises at late G1and increases steadily until the late G2 phase of the cell cycle. Afterwards, it disappears slightly ahead of Cyclin B1 during mitosis which is compatible with its activity as upstream activator of mitosis entry [41, 42]. When Cyclin A was assessed, we observed a significant decrease after 15 and 17h T44Bf treatment for all the cell lines showing that arrest observed at G2/M by flow cytometry corresponds to cells at mitosis rather than at the G2 phase of the cell cycle (Fig 9A).

The regulatory events responsible for G2 to M phase transition involve the activation of the Cyclin B1-Cdc2 kinase complex, also called M phase-promoting factor (MPF) [43, 44]. When Cyclin B1 levels were analyzed by western blot assay, we observed that T44Bf increased Cyclin B1 after 15, 17 and 20h of treatment in all cell lines consistent with the mitotic arrest at prometaphase observed by both flow cytometry and IF assays (Fig 9B). It is worth noting that similar results were observed when Paclitaxel, a prototypical microtubule inhibitor, was tested as a positive control that also induces G2/M arrest, as confirmed by flow cytometry (data not shown). When total Cdc2 levels were assessed, no significant differences were observed between treated and untreated cells, in accordance with the fact that Cdc2 levels are relatively constant throughout the cell cycle and its activity regulation is mainly at the post-translational level (Fig 9B).

In agreement with the increase in the G0/G1 subpopulation observed in the reversion assay, Cyclin B1 levels significantly decreased after 2h and 5h following T44Bf replacement by fresh medium suggesting that T44Bf impairs anaphase progression (Fig 9B). This is consistent with the fact that defects in the proper alignment of chromosomes induce permanent activation of mitotic checkpoint by inhibiting Cyclin B1 degradation [45, 46]. Once SAC requirements are satisfied, anaphase onset depends on the loss of MPF activity which is associated to Cyclin B1 subunit degradation. Therefore, these results indicate that T44Bf treatment promotes prometaphase arrest in a reversible manner probably by activating the mitotic checkpoint, which is consistent with the downregulation of Cyclin A and the modulation of Cyclin B1 levels observed both, in the presence of T44Bf and following its removal.

## Discussion

In this work, we describe a novel activity for a benzophenone thiosemicarbazone, T44Bf, a compound that exhibits anti-leukemic properties. T44Bf significantly reduces cell proliferation in a concentration- and time- dependent manner not only in AML models, as the KG1a (M0), HL60 (M2-3) and U937 (M5) cell lines, but also in a model of ALL like the Jurkat cell line. In addition, our study indicates that programmed cell death is a consequence of growth inhibition induced by T44Bf. It is worth noting that although the AML models used in the present work belong to different stages of hematopoietic cell differentiation and linage, all of them responded to T44Bf treatment. However, differences among maximal proliferation inhibition, cell cycle arrest and apoptotic activity were evidenced in the more undifferentiated cell line KG1a and the lymphoid Jurkat cells. Depending on the cell type and stimuli, many previous studies have associated ERK activity to antiproliferative events such as apoptosis [33–35]. Our results show that T44Bf treatment induces an early- and late-sustained phosphorylation of the MAPK ERK1/2. This event proved to be a necessary step to achieve the pro-apoptotic effect of T44Bf, as shown by the absence of caspase 3 and PARP-cleaved fractions in presence of the MEK inhibitor U0126. Interestingly, the pro-apoptotic activity of T44Bf showed high selectivity for leukemia cellular models respect to normal PBMC, since both resting and proliferating PBMC were resistant to T44Bf-induced apoptosis. This finding represents an important feature in drug development of a pharmacological prototype. Some of the adverse effects associated to agents such as cytarabine and anthracyclines involve acute myelosupression and cardiotoxicity [6, 47]. In this sense, T44Bf would show an advantageous feature respect to currently used agents for the treatment of AML.

In normal cell cycle progression, the signaling pathway responsible for mitotic entry depends on MPF activation. An upstream regulator is the protein Cyclin A, that increases steadily until late G2 phase, but it is no longer required after MPF activation [41]. Indeed, Cyclin A begins to disappear shortly after nuclear envelope breakdown, ahead of Cyclin B1 and its degradation is independent of mitotic checkpoint or SAC [48–50]. Afterwards, mitotic exit from metaphase involves the briefly activated SAC. However, upon the presence of defects in the proper alignment of chromosomes, the mitotic checkpoint cannot be satisfied, leading cells to a chronic mitotic arrest and eventually apoptosis with the aim of preventing proliferation of damaged cells [51]. SAC acts mainly through the regulation of Cyclin B1 degradation, but not Cyclin A, [49], maintaining high levels of Cyclin B1 during the arrest by E3 ubiquitin ligase inhibition.

Once chromosomes are properly attached and oriented, SAC is turned off and Cyclin B1 degraded allowing the onset of anaphase [51, 52]. In this context, anti-mitotic compounds cause a mitotic arrest either by interfering with microtubules dynamics or inducing DNA damage in p53-deficient tumor cells [9, 53]. Flow cytometry, immunofluorescence and Cyclin A western blot analysis, showed that T44Bf induces arrest of cells at prometaphase rather than G2 phase, with a concomitant increase in Cyclin B1 protein levels, thus suggesting that SAC requirements are not satisfied.

Moreover, T44Bf withdrawal allowed cell progression to G0/G1 along with decreased levels of Cyclin B1, supporting that T44Bf-induced arrest is a reversible event. This observation (supported by the cell cycle assay and further confirmed by Cyclin A and Cyclin B expression), may be an advantageous feature as regards the toxicity exerted by diverse chemotherapeutic agents. In this sense, it has been described in the literature that although the efficacy of treatments with drugs with reversible activity like Vincristine, improves with time exposure, symptoms due to toxicity are reduced a few weeks after drug withdrawal [54–56]. We think that this is a very important aspect as regards the quality of life of patients. In addition the reversibility of T44Bf action suggests that this compound may act by binding in a non-covalent manner to a specific target, which probably results in an impairment to satisfy SAC requirements for anaphase progression. However, in these cases it is important to consider the bioavailability of these compounds to achieve an adequate treatment regimen. Combinatorial drug therapy has shown to increase the efficacy of anti-mitotic compounds. Considering the high sensitivity exhibited by cells arrested at mitotic checkpoint, simultaneous treatment with agents-targeting different stages of the cell cycle could help to circumvent therapy resistance development and to maximize the apoptotic response [57, 58].

The chronic arrest of cells at the mitotic phase is usually followed by an apoptotic response associated to the mitochondria signaling pathway [59]. Some common events associated to this pathway relate to changes in the expression and post-transcriptional modifications of anti-apoptotic (Bcl-2 and Bcl-xL) and pro-apoptotic (Bad and Bax) proteins, as well as the loss of inner mitochondrial membrane integrity [60]. The data presented here shows that T44Bf-induced apoptosis involves the loss of mitochondrial membrane potential, a time-dependent increase of phosphorylated Bcl-xL fraction, as well as an increase in Bcl-2 and total Bad levels. Also a Bcl-2 phosphorylated fraction, evidenced by a mobility shifted band, was induced after T44Bf treatment. These results indicate the activation of the mitochondria signaling pathway in relation to apoptosis and the loss of normal mitochondrial function. Several lines of evidence show that while Bcl-2 phosphorylation may be related to either protein activation or inactivation, it could also be associated to normal cells in G2/M phase [31, 61]. In this sense, some reports identified Bcl-2, Bcl-xL and Bad as Cdc2-cyclin B1 targets in G2/M both in normal and arrested cells [62–66]

Until now, most aromatic TSCs with pharmacological effects in cancer display similar mechanisms of action, such as inhibition of ribonucleotide reductase and topoisomerase II alpha, and generation of intracellular ROS [67–71]. All these mechanisms are intrinsically related to their ability to act as strong chelators of transition metals such as Fe(II), Cu(II), and Zn(II) through their inherent N-N-S tridentate coordination scaffold [72, 73]. In the particular case of T44Bf such scaffold is not present, since it has only two potential coordination atoms (N1 and S in the thiosemicarbazone moiety), thus the chelation of transition metals is not expected to occur. On the other hand, a series of benzophenone TSC analogs have been developed as cathepsin L inhibitors with a potential application as therapeutic agents against cancer metastasis [74]. These TSCs differ from T44Bf in the presence of strong electronegative substituents mainly in *meta-* position of one or both aromatic rings, sometimes in combination with another strong electron withdrawing atom or group in *orto-* or *meta-* position to yield the most active compounds of the series [75, 76]. This substitution pattern in the benzophenone moiety has been recently proven essential for cathepsin L inhibition [77]. Since T44Bf is *para*-methoxy disubstituted, it is not likely to act as the other benzophenone derivatives described. Although further studies are needed to unveil its specific target it may be proposed that T44Bf mechanism of action relies on the disruption of normal cell cycle progression.

**Fig 10:**
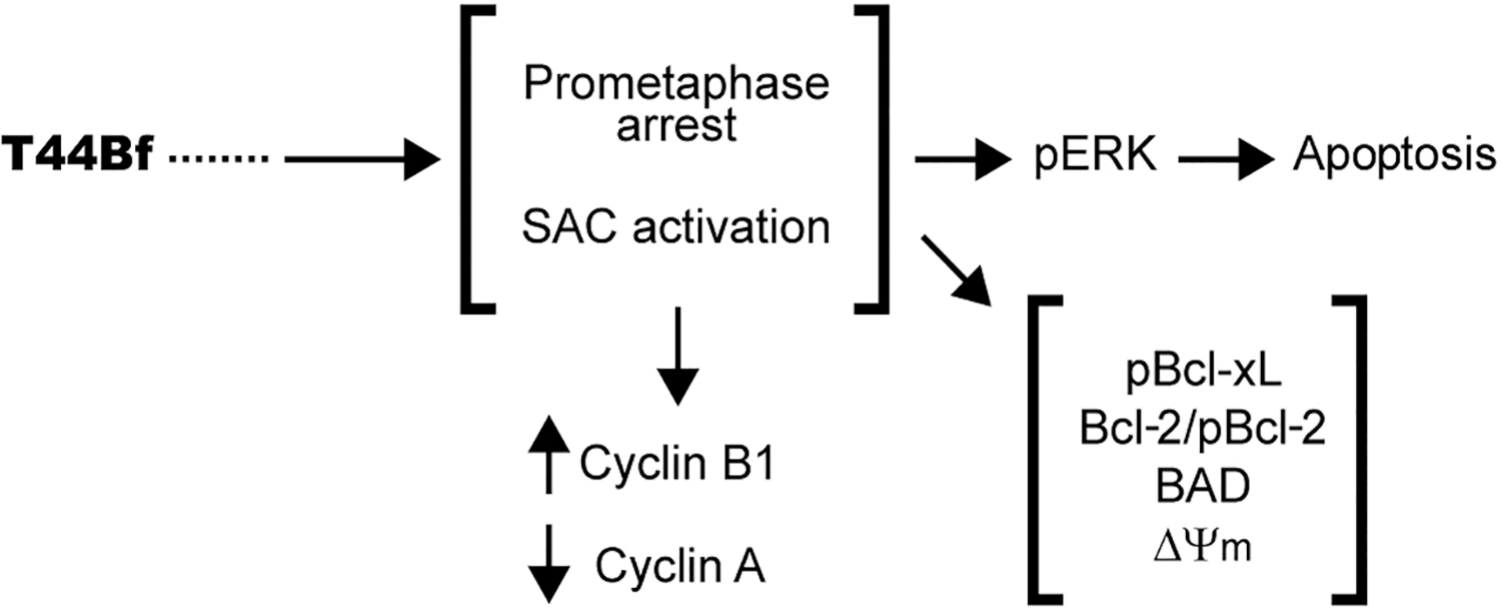
Schematic diagram of T44Bf mechanism of action in human acute leukemia cells. T44Bf induces chronic prometaphase arrest, associated to SAC permanent activation through a currently unknown mechanism. This is evidenced by Cyclin A downregulation, increased Cyclin B1 levels and condensed chromosomes visualization. This chronic arrest leads to an apoptotic response involving mitochondrial signaling pathway activation and up-regulation of ERK pathway, which is a requisite for T44Bf-induced cell death.

In summary, our study suggests a new activity for a benzophenone thiosemicarbazone with high potential to be developed as an anti-leukemic agent for acute leukemia treatment. The selectivity exhibited, in addition to the mechanism of action involved (Fig 10) makes of T44Bf a good candidate for preclinical research and encourages further *in vivo* studies. Also, combinatorial treatment assays seem to be a promissory alternative for future studies involving T44Bf.
